# 

**Published:** 1845-12

**Authors:** 


CONTENTS:

Case of complete inversion of the Uterus, by E. FISHER, M. D. 129
Verminous Irritation, a direct exciting cause of Diseases not usually attributed to any of the varieties of Intestinal Worms, by DR. J. C. SCOTT. - - 132
Proposed National Convention. 136
Rush Medical College. - - 138

BIBLIOGRAPHICAL NOTICES.

The Domestic Management of the Sick Room, necessary, in aid of Medical treatment, for the cure of diseases, by ANTHONY TODD THOMPSON, M.D., F. L. S. &c. 139
The Anatomical Remembrancer, or complete Pocket Anatomist. 140
The Medical Remembrancer, or Book of Emergencies, by EDWARD B. S. SHAW, M. R. C. S., & L. A. S., &c. &c. - 140

PRACTICAL MEDICINE, &c.

On the Endermic use of Purgatives 141
On the Diagnosis of Fracture. 141
On the Pathology of Toothache. 142
Notice to Readers and Correspondents. - - - - 144

